# Sjögren’s syndrome and acquired splenic atrophy with septic shock: a case report

**DOI:** 10.1186/1752-1947-8-10

**Published:** 2014-01-06

**Authors:** Natacha Santos, Rui Silva, Joana Rodrigues, José Torres-Costa

**Affiliations:** 1 Allergy and Clinical Immunology Division, Centro Hospitalar São João, EPE: Alameda Professor Hernâni Monteiro, 4202-451 Porto, Portugal; 2 Allergy and Clinical Immunology Unit, Centro Hospitalar de Trás-os-Montes e Alto Douro, EPE: Av. Noruega, Lordelo, 5000-508 Vila Real, Portugal; 3 Cardiology Division, Centro Hospitalar São João, EPE: Alameda Professor Hernâni Monteiro, 4202-451 Porto, Portugal

**Keywords:** Asplenia, Hyposplenism, Immunodeficiency, Septic shock, Sjögren’s syndrome, Splenic atrophy

## Abstract

**Introduction:**

The most frequent causes of adult-onset recurrent infections are human immunodeficiency virus infection, malignancy, and autoimmune diseases, while acquired non-surgical hyposplenism is rare. Although acquired asplenia/hyposplenism have been described in association with celiac disease and, less frequently, with autoimmune diseases such as Sjögren’s syndrome, the manifestations in this context are usually only detectable in the laboratory setting, with Howell-Jolly bodies or thrombocytosis. To the best of our knowledge, no previous case of pneumococcal septic shock in a patient with acquired hyposplenism and co-morbid Sjögren’s syndrome has been reported.

**Case presentation:**

We report a case of a 45-year-old Caucasian woman who developed pneumococcal pneumonia at age 42 years, pneumococcal meningitis at age 44 years and septic shock with *Streptococcus agalactiae* bacteremia at age 45 years and was subsequently diagnosed with radiological splenic atrophy and functional asplenia, as well as primary Sjögren’s syndrome. After appropriate immunizations, the patient has been free from clinically important infections.

**Conclusion:**

Hyposplenism should be suspected in patients with adult-onset infections caused by encapsulated bacteria, especially if autoantibodies are present. Early diagnosis can help prevent potentially life-threatening infections. Possible associations between splenic atrophy and Sjögren’s syndrome are discussed.

## Introduction

The most frequent causes of adult-onset recurrent infections are primary antibody deficiencies, such as common variable immunodeficiency, and secondary states of immunodeficiency, such as human immunodeficiency virus (HIV) infection, malignancy or autoimmune diseases. Acquired, non-surgical, functional asplenia is a rare cause [1]. Although acquired asplenia and/or hyposplenism have been associated with celiac disease [2] and, less frequently, with autoimmune diseases, such as Sjögren’s syndrome [3], the manifestations of hyposplenism in this context are usually restricted to laboratory findings such as Howell-Jolly bodies or thrombocytosis. Septic shock has been documented in celiac disease–associated splenic atrophy, especially in adult-diagnosed celiac disease with associated cavitating mesenteric lymph node syndrome [2]. To the best of our knowledge, no case of pneumococcal septic shock in a patient with acquired splenic atrophy and co-morbid Sjögren’s syndrome has previously been reported.

## Case presentation

A 45-year-old Caucasian woman was referred to the Division of Allergy and Clinical Immunology at our institution for severe, recurrent, adult-onset infections. Her previous medical history of infections included tuberculous cervical lymphadenitis at age 33 years, community-acquired pneumococcal pneumonia at the age of 42 and a 2-week stay in the Division of Infectious Diseases at our hospital at age 44 for bacterial meningitis with *Streptococcus pneumonia* bacteremia, which was treated with a 12-day course of intravenous ceftriaxone. At the age of 45, she had been hospitalized for treatment of septic shock with refractory hypotension and a need for assisted ventilation. She had presented earlier the same day with a tympanic temperature of 39°C and malaise. Her leukocyte count was 45 × 10^9^/L with 90% neutrophils. A blood smear revealed Howell-Jolly bodies and target cells. Her C-reactive protein level was 288mg/dl. Her blood cultures were positive for *Streptococcus agalactiae*. She remained hospitalized for 2 months because of several complications, which included hypokalemia-induced cardiorespiratory arrest; large-volume bilateral pleural effusions; bilateral multifocal pyelonephritis; iatrogenic subcapsular hematoma after liver biopsy; a second episode of septic shock; nosocomial colonization of bronchial secretions and infection with *Acinetobacter baumannii*, *Pseudomonas aeruginosa* and *Staphylococcus aureus*; and urine cultures positive for *Enterococcus faecalis* and *Pseudomonas aeruginosa*. She was initially treated with ceftriaxone and ampicillin, but other antibiotics were later added for treatment of the nosocomial infections.

The patient’s personal medical history included late menarche at age 18 and early menopause at age 33, which she had been told was iatrogenic due to taking anti-tubercular drugs. She had no history of pregnancy. Other relevant medical history included degenerative disc disease and smoking habits. She denied having previous surgeries, taking recent trips to tropical countries, drug abuse or high-risk sexual habits. She was receiving hormone replacement therapy. Her family history was unremarkable except for her father, who had died at the age of 52 years due to a stroke. She had no known family history of primary immunodeficiencies.

**Figure 1:**
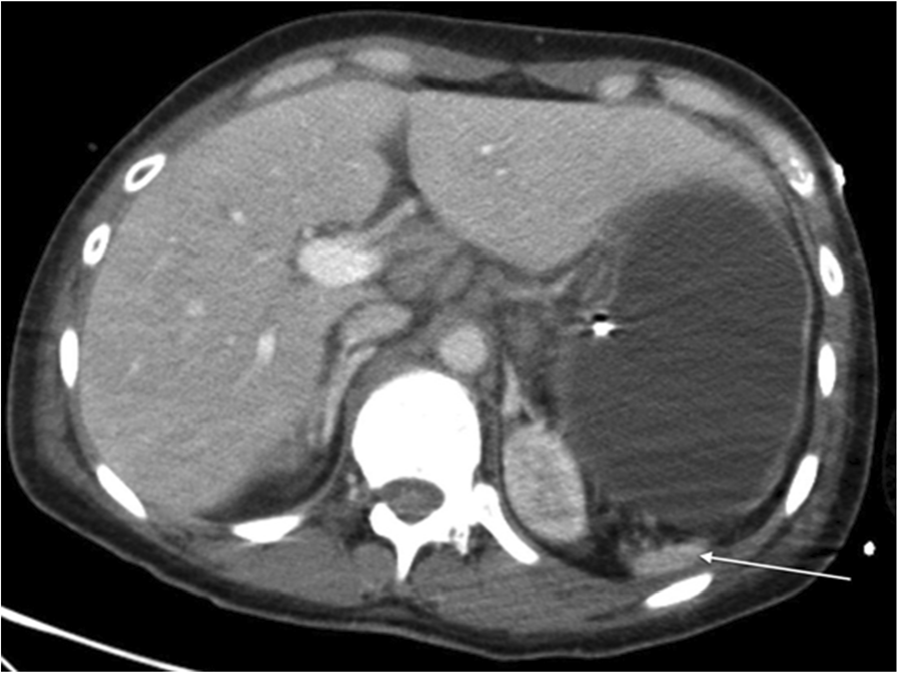
Computed tomography of the abdomen showing splenic atrophy (arrow).

The patient’s clinical examination was unremarkable. She had previously undergone an immunological work-up 7 months before hospitalization to exclude primary antibody deficiency and autoimmune diseases. Her immunoglobulin (Ig) levels were normal (reference ranges in parentheses): IgG, 14.8g/L (7.0g/L to 16.0g/L); IgG1, 13 (4.1g/L to 11.4g/L); IgG2, 1.49g/L (1.5g/L to 6.4g/L); IgG3, 0.59g/L (0.20g/L to 1.10g/L); IgG4, 0.117g/L (0.080g/L to 1.40g/L); IgA, 2.10g/L (0.7g/L to 4.0g/L); and IgM, 1.93g/L (0.4g/L to 2.3g/L). Tests were negative for circulating immunocomplexes and the following antibodies: anti-nuclear antibody (ANA), anti-double-stranded deoxyribonucleic acid (DNA) (anti-dsDNA), anti-thyroglobulin and anti-peroxidase. Her rheumatoid factor level was elevated (818IU/L; normal range, <30IU/L). Tests for HIV and hepatitis B and C virus were negative. Other investigations performed during her hospital stay included computed tomography of the abdomen, which revealed splenic atrophy (Figure 1), and a technetium-99 scan, which showed no functional splenic tissue in the usual location and no ectopic spleen. A thorough investigation for malignant diseases was performed, including liver biopsy, pleural effusion cytology and pleural biopsy, ultrasonography of the neck and supraclavicular fossae, biopsy of a cervical lymph node, uterine cervix cytology, and mammography, which were all negative. A bone marrow biopsy and blood immunophenotyping were inconclusive, showing dysmorphic cells in the megakaryocyte lineage. Her rheumatoid factor level remained elevated during hospitalization with 335IU/L and, later, 531IU/L. An immunologic evaluation revealed positive, speckled ANAs (1:320), positive anti-extractable nuclear antigen antibodies directed against SSA and polyclonal hypergammaglobulinemia (25g/L). Tests for anti-cyclic citrullinated peptide, anti-dsDNA, anti-gliadin and anti-transglutaminase antibodies were negative.

After her hospital discharge, she received the 23-valent pneumococcal vaccine, as well as immunization for *Neisseria meningitidis* and *Haemophilus influenzae* type b. After a thorough functional inquiry, the patient reported having dry eyes. She denied xerostomia or any cutaneous, gastrointestinal or articular complaints. After complete recovery from her infections, her leukocyte count was 12 × 10^9^/L with lymphocytosis (7 × 10^9^/L) and monocytosis (1.2 × 10^9^/L). Her platelet count was normal. Her rheumatoid factor level was 2950IU/L. The tear film break-up time with fluorescein was longer than 10 seconds, which is consistent with keratoconjunctivitis sicca. A biopsy of a minor salivary gland confirmed lymphocytic sialadenitis with two foci of lymphocytes per 4mm^2^, which is consistent with a diagnosis of Sjögren’s syndrome. She continued hormone replacement therapy and was also prescribed artificial tears for daily use. Her anti-pneumococcal antibody levels were normal (reference levels in parentheses): IgG, 10.7mg/dl (>1.54mg/dl); and IgG2, 3.05mg/dl (>0.54mg/dl). At the time of her 2-year follow-up examination, she had not experienced further infectious events.

## Discussion

Two unusual diagnoses were made for the patient reported herein. First, splenic atrophy, a rare condition, was diagnosed as the cause of her recurrent, adult-onset infections. Second, splenic atrophy was co-morbid with Sjögren’s syndrome, which is a rare association. To the best of our knowledge, this report is the first one to describe such a dramatic clinical presentation.

The spleen has several functions, including removal of defective blood cells and poorly opsonized bacteria, such as encapsulated organisms; synthesis of IgM and opsonins such as tuftsin and properdin; and maintaining the survival of IgM memory B cells [4]. The textbook clinical phenotype of hyposplenism is overwhelming pneumococcal sepsis [4]. Hyposplenism can occur either in the context of splenic atrophy or when the spleen is anatomically present or even enlarged (that is, functional hyposplenism), and it can be diagnosed on the basis of absent uptake of Tc-99 sulfur colloid. Leukocytosis, lymphocytosis, monocytosis and thrombocytosis can provide clues to the diagnosis, and red cell abnormalities in the peripheral blood smear, such as acanthocytes, target cells (codocytes), Heinz bodies, Pappenheimer bodies, and especially Howell-Jolly bodies, are usually present [4]. The most common cause of hyposplenism is surgical splenectomy, with other common causes being celiac disease, sickle hemoglobinopathies, alcoholism, bone marrow transplantation and systemic lupus erythematosus (SLE). An association with several autoimmune disorders, including Sjögren’s syndrome, has also been described previously [4]. Sjögren’s syndrome is a chronic inflammatory disease characterized by lymphocytic infiltration and impaired function of the lacrimal and salivary glands. The main manifestations are keratoconjunctivitis sicca and xerostomia, although lymph node and salivary gland enlargement, non-Hodgkin’s lymphoma, myalgia, fatigue and cognitive dysfunction may also occur [5]. New diagnostic criteria proposed in 2012 by the American College of Rheumatology [6] include at least two of the following: (1) positive serum anti-SSA and/or anti-SSB antibodies or positive rheumatoid factor and ANA titers 1:320 or higher, (2) a Sjögren’s International Collaborative Clinical Alliance ocular staining score of 3 or higher and (3) lymphocytic sialadenitis with one or more foci of 50 lymphocytes per 4mm^2^. All three of these criteria were present in our patient.

This combination of diagnoses was previously reported in two patients investigated for thrombocytosis, who were found to have functional hyposplenism and Sjögren’s syndrome [3], but, to the best of our knowledge, splenic atrophy and septic shock in a patient with Sjögren’s syndrome have not been reported previously. Nevertheless, a causal relationship between these two entities cannot be proved. On the one hand, autoantibodies occur in 26% to 38% of patients who have undergone splenectomy, possibly due to a reduction in the number of suppressor T cells, although development of active autoimmune disease has not been reported [7,8]. On the other hand, several antibody-mediated diseases, such as celiac disease and SLE, have been associated with the development of functional hyposplenism or even splenic atrophy, with possible mechanisms including the production of fibrotic factors by self-reactive lymphocytes and Fc receptor blockade by circulating immune complex saturation [3,4]. Because no other causative explanation for our patient’s splenic atrophy was identified, it is possible that long-standing undiagnosed Sjögren’s syndrome progressively caused loss of splenic function and increased her susceptibility to pneumococcal infections, ultimately leading to splenic atrophy and the septic shock event.

## Conclusion

We describe a case of a patient with splenic atrophy and its possible association with Sjögren’s syndrome. Hyposplenism should be included in the differential diagnosis of patients with recurrent pneumococcal infections when other frequent causes have been ruled out, especially if autoantibodies are present, because early diagnosis and adequate immunization can help prevent potentially life-threatening infections.

## Consent

Written informed consent was obtained from the patient for publication of this case report and any accompanying images. A copy of the written consent is available for review by the Editor-in-Chief of this journal.

## Abbreviations

ANA: Anti-nuclear antibody; SLE: Systemic lupus erythematosus.

## Competing interest

The authors declare that they have no competing interests.

## Authors’ contributions

All authors contributed directly to the investigation of the clinical case. NS wrote the manuscript. All authors reviewed the manuscript and gave their approval for the final version to be published.
